# Using High-Throughput Amplicon Sequencing to Evaluate Intragenomic Variation and Accuracy in Species Identification of *Cordyceps* Species

**DOI:** 10.3390/jof7090767

**Published:** 2021-09-16

**Authors:** Soumitra Paloi, Wuttichai Mhuantong, Janet Jennifer Luangsa-ard, Noppol Kobmoo

**Affiliations:** National Center for Genetic Engineering and Biotechnology (BIOTEC), National Science and Development Agency (NSTDA), 113 Thailand Science Park, Phahonuyothin Rd., Khlong Nueng, Khlong Luang, Pathum Thani 12120, Thailand; soumitrabotany@gmail.com (S.P.); wuttichai.mhu@biotec.or.th (W.M.); jajen@biotec.or.th (J.J.L.)

**Keywords:** barcoding, *Cordyceps*, intragenomic variation, nrDNA, PacBio sequencing

## Abstract

While recent sequencing technologies (third generation sequencing) can successfully sequence all copies of nuclear ribosomal DNA (rDNA) markers present within a genome and offer insights into the intragenomic variation of these markers, high intragenomic variation can be a source of confusion for high-throughput species identification using such technologies. High-throughput (HT) amplicon sequencing via PacBio SEQUEL I was used to evaluate the intragenomic variation of the ITS region and D1–D2 LSU domains in nine *Cordyceps* species, and the accuracy of such technology to identify these species based on molecular phylogenies was also assessed. PacBio sequences within strains showed variable level of intragenomic variation among the studied *Cordyceps* species with *C. blackwelliae* showing greater variation than the others. Some variants from a mix of species clustered together outside their respective species of origin, indicative of intragenomic variation that escaped concerted evolution shared between species. Proper selection of consensus sequences from HT amplicon sequencing is a challenge for interpretation of correct species identification. PacBio consensus sequences with the highest number of reads represent the major variants within a genome and gave the best results in terms of species identification.

## 1. Introduction

After the discovery of polymerase chain reaction (PCR) technique and Sanger sequencing in the early 90s [1], nucleotide states became important characters in fungal taxonomy and molecular markers have been continuously applied in this field [2,3]. Nowadays, molecular markers are regularly used for delimiting novel species, identification, and the inference of phylogenetic relationship. The nuclear internal transcribed spacer (nrITS) regions of the ribosomal DNA (rDNA) is used as a universal barcoding region, because a broad range of fungi can be identified using this region [4]. nrITS is still the most widely used for species identification, barcoding and fungal phylogenetics [3,5], although many studies have shown that its utility for species identification was limited [6,7,8]. Another commonly used ribosomal marker is the Ribosomal Large Subunit (nrLSU). Particularly, the D1–D2 variable domains showed sufficient power for identification at the genus level and has been successfully used in fungal taxonomy, including the Assembling the Fungal Tree of Life (AFTOL) Project [9,10,11]. The nrLSU region generally reflects a smaller amount of variation than nrITS [12]. Both regions have some strength and restrictions for fungal identification and taxonomic classification while ITS-D1–D2 LSU combined regions have been shown to demonstrate better performance [13,14]. With a large number of rDNA copies in the genome, both ITS and LSU allow easy PCR amplification with a very small amount of DNA [8,15,16]. This advantage comes with a cost. The variation found in an individual genome, e.g., intragenomic variation, can hamper identification and molecular systematics. Although concerted evolution generally maintains the intragenomic variation to a minimal level, within-genome copies can undergo pseudogenization [17] or recombination with inter-fertile closely related species [18,19].

The intragenomic variation of rDNA can be perceived through the occurrence of double or multiple peaks in chromatograms following direct sequencing of PCR products containing multiple amplicons. Sanger sequencing can sometimes result in ambiguous sequences due to polymorphisms between amplicons [20]. Recent technologies in high-throughput sequencing can allow taxonomists to better address intra-genomic variation (allelic variation) via high-throughput amplicon-sequencing (HT amplicon-seq). Studies in other organisms such as insects showed a better taxonomic resolution at the species level using HT amplicon-seq of ITS2 than Sanger sequencing [21]. Such studies in fungi are still rare [22]. The high-throughput nature of recent technologies may be problematic as this technology can sequence all types of variation which end up confusing taxonomists. It is essential to evaluate, on the one hand, the level of intragenomic variation among organisms of interest, and on the other, the capacity of HT amplicon-seq to identify and classify species correctly.

As part of a project aiming at evaluating the performance of HT amplicon-seq with PacBio sequencing in the identification and classification of entomopathogenic fungi from our collections, we detailed here results based on PacBio amplicon sequencing of the ITS region (ITS1-5.8S-ITS2) and D1–D2 LSU domains from the genus *Cordyceps* (Cordycipitaceae, Hypocreales). *Cordyceps* is the most diverse genus of insect pathogenic fungi. Until now, more than 280 species have been documented from different parts of the world [23]. The genus *Cordyceps* is the type genus of the family Cordycipitaceae [24]. Tropical and sub-tropical regions show the highest known species diversity, particularly in east and south-east Asia [24]. The genus *Cordyceps* does not have distinctive unique characteristics that discriminate it from closely related genera such as *Blackwellomyces* and *Samsoniella* [25]. *Cordyceps* species are parasitic on insects of various orders including Coleoptera, Diptera, Hemiptera, Hymenoptera, Lepidoptera, Orthoptera and also on spider [25]. Therefore, their identification relies mainly on molecular tools. Due to its high diversity, species identification in *Cordyceps* requires sufficiently polymorphic regions such as ITS. In this study, we generated sequences from PacBio Sequel I of the ITS region combined with D1–D2 LSU domains from nine *Cordyceps* species including *C. blackwelliae, C. cateniannulata, C. chiangdaoensis, C. javanica, C. kuiburiensis, C. lepidopterorum*, *C. morakotii*, *C.* cf. *ninchukispora*, and *C. tenuipes* based on specimens already identified with accuracy from previous studies [25,26,27,28]. The data allowed us to evaluate the level of intragenomic variation via analyses of sequence divergence and haplotype network. We opted for an amplicon sequencing-based approach, rather than a metagenomics approach, as we were interested in identifying well-curated cultures and specimens but not environmental samples. Furthermore, the metagenomics would not allow a transparent access to intragenomic variation of rDNA. PacBio technology was used in this study because of its long reads that allow an instant sequencing of both ITS and D1–D2 regions, and thus do not require any assembly step to reconstruct the whole regions such as short-read sequencing (e.g., Illumina) that would introduce additional errors [29]. We compared the PacBio sequences to those obtained using Sanger sequencing to assess whether the variants revealed with the PacBio technology could identify the specimens to the species level correctly based on molecular phylogenies.

## 2. Materials and Methods

### 2.1. The Sample Collection and DNA Extraction

**Figure 1:**
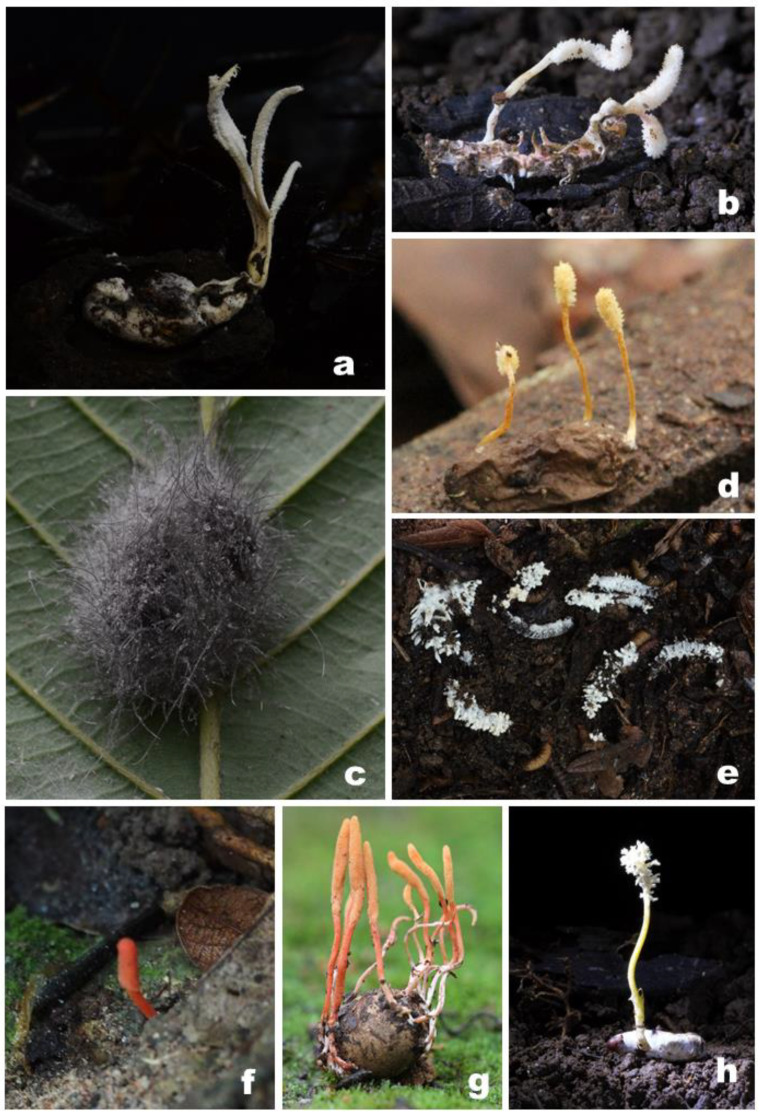
Field photographs of some Cordyceps species: (**a**) *C. blackwelliae*; (**b**) *C. chiangdaoensis*; (**c**) *C. javanica*; (**d**) *C. morakotii*; (**e**) *C. lepidopterorum*; (**f**) *C. kuiburiensis*; (**g**) *C.* cf. *ninchukispora*; (**h**) *C. tenuipes*.

A total number of 22 strains belonging to nine *Cordyceps* species (Figure 1) were selected from the BIOTEC Culture Collection (BCC) with deposited specimens in the BIOTEC Bangkok Herbarium (BBH); duplicate cultures also exist at the Thailand Bioresource Research Center (TBRC). Genomic DNA was extracted from mycelia obtained from cultures on PDA using a slightly modified cetyl-trimethyl ammonium bromide (CTAB) method, described in Mongkolsamrit et al. [30]. Briefly, fungal mycelia (5 to 10 mg) from culture plate were harvested into a 2 mL Eppendorf. 600 μL of pre-heated CTAB buffer were added and the mycelia were grinded with a pestle. After vortexing and an incubation at 65 °C for one hour, 700 μL of CIAA (Chloroform:Isoamylalcohol 24:1) were then added for protein precipitation. The preparation was then vortexed and centrifuged at 13,000 rpm, 25 °C for 10 min. The supernatant was pipetted into a new 1.5 mL Eppendorf tube. 600 μL of cold isopropanol were then added, mixed and incubated in ice for 30 min to precipitate the DNA. Finally, the preparation was centrifuged at 13,000 rpm, for 20 min and the supernatant was discarded. The DNA pellet was washed with 70% ethanol and air-dried. The DNA was dissolved in 100 μL of TE buffer and stored at −20 °C.

### 2.2. PacBio Amplicon Sequencing

PCR amplifications for the whole nrITS region with the D1–D2 domains of nrLSU were carried out simultaneously using specifically designed primers in which the ITS5 (forward: GGAAGTAAAAGTCGTAACAAGG) [31] and LR5 (reverse: TCCTGAGGGAAACTTCG) [15] primers were each tagged with a different barcode sequence, resulting in different combinations corresponding to distinct PCR reactions. The amplifications were conducted on an 2720 automated thermal cycler (Applied Biosystems, Waltham, MA, USA). A hot start of 4 min at 94 °C was followed by 30 cycles consisting of 3 min at 94 °C, 1 min at 50 °C, 2 min at 72 °C, and a final elongation step of 3 min at 72 °C, using Dream Taq DNA polymerase (Thermo Fisher, Waltham, MA, USA). Another set of PCR for the same strains were carried out with Platinum SuperFi DNA polymerase (Invitrogen, Waltham, MA, USA) using the same PCR protocol as above. This latest polymerase has >300× fidelity to the Dream Taq. The objective was to assess the difference in amplification and phylogenetic identification between a high-fidelity polymerase and a standard Taq. PCR products from both polymerases were purified using an AMPure XP DNA purification kit. DNA concentration of the purified products was quantified using Qubit^TM^. All purified PCR products were adjusted to the same concentration of approximately 15 ng/µL. The pooled amplicons were sent to OmicsDrive (Singapore) for a sequencing with a PacBio SEQUEL I machine. 

Once the raw data were obtained, Circular Consensus Sequences (CCS) were determined from subread sequences by CCS tool [32] using a required minimum of five subreads and read quality (rq) of at least 0.99 (>99% accuracy). The sequence of each sample was demultiplexed from its barcodes using custom Python script (Python version 3.7, scikit-bio package version 0.5.5). The sample barcodes were not allowed to have more than three mismatches. Only sequences with a length between 1000 and 2000 bp were kept. All sequences were bioinformatically cleaved between the ITS and D1/D2 LSU regions using the ITS4 priming sites to cut through. In each sample, sequences were clustered by CD-HIT-EST [33] at 97% similarity, then sequences in each cluster were aligned by MUSCLE [34] and a consensus sequence was generated per cluster. The raw PacBio reads from the project and ITS and D1/D2 LSU barcodes obtained were deposited at the Mendeley Data Repository [35]. 

### 2.3. Sanger Sequencing

Most of the specimens already had Sanger sequences (nrITS and nrLSU) deposited in NCBI Genbank from previous studies on *Cordyceps* species (Table 1) [25,26,27,28] while four strains lacked ITS or LSU Sanger sequences. PCR amplifications were thus carried out for these strains following [27], using the universal primers ITS5 and ITS4 for the whole ITS1-5.8S-ITS2 region and LROR-LR7 for the D1/D2 domain of LSU [31]. PCR products were purified using QIAquick^®^ Gel Extraction Kit (QIAGEN, Hilden, Germany) and were subjected to automated DNA sequencing on an ABI3730xl DNA Analyzer (Applied Biosystems), using the same primers. The generated sequences were then deposited in NCBI GenBank (Table 1). For the purpose of phylogenetic classification, other nrITS and nrLSU sequences from *Cordyceps* (Supplementary Table S1), also generated from Sanger sequencing, were included [25,27]. 

### 2.4. Phylogenetic Analyses

The consensus sequences derived from each PacBio clusters and sequences through Sanger sequencing were aligned together. The Sanger sequences were used to evaluate the PacBio sequences. For an ITS-LSU combined analysis, the PacBio-based consensus sequences were selected only from the clusters with the highest number of CCS. For all datasets, two species, *Blackwellomyces calendulinus* (ITS: MT000695; LSU: MT003031) and *B. aurantiacus* (ITS: MT000692; LSU: MT003028) were used for rooting the phylogenies. Data were separated into two datasets following the type of polymerase (Dream Taq vs. SuperFi polymerases), in order to see whether both polymerases gave different outcomes to the phylogenetic classification.

Sequences were aligned by ClustalX2 [36] with default settings. All the phylogenetic analyses were conducted using CIPRES web portal [37]. Maximum likelihood (ML) and Bayesian inference (BI) were performed using the GTR + G, GTR + I + G and GTR + I + G model as selected by jModeltest 2.1.6 [38], for nrITS, nrLSU and combined dataset, respectively. ML analyses were carried out using RAxML-v. 8.2.9 with 1000 bootstrap replicates [39]. BI analyses were carried out using Metropolis-coupled Markov chain Monte Carlo (MCMCMC) methods via Mr. Bayes v. 3.2.2 [40]. The Markov chains were run for 10^6^ generations, saving a tree every 100th generation. Default settings in Mr. Bayes were used for the incremental heating scheme for the chains (3 heated and 1 cold chain), unconstrained branch length (unconstrained: exponential (10.0)), and uninformative topology (uniform) priors. Mr. Bayes was used to compute a 50% majority rule consensus of the remaining trees after 25% burn-in phase, to obtain estimates of posterior probabilities (PPs).

### 2.5. Sequence Divergence Analysis

**Figure A1:**
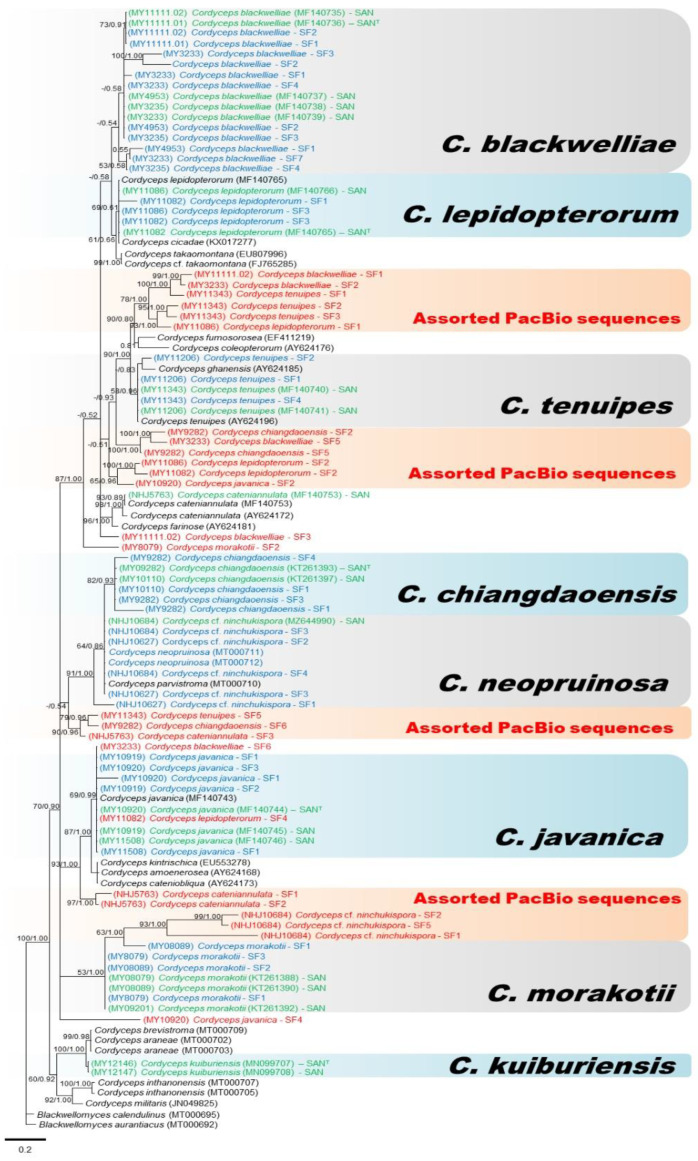
Consensus phylogram (50% majority rule) from a Bayesian analysis of the nrITS sequences (obtained from SuperFi DNA polymerase), following an MCMC analysis of 10^6^ generations. Maximum likelihood bootstrap values >50% (left of/) and Bayesian posterior probabilities >0.50 (to the right). The scale bar represents number of expected changes per site. Some PacBio cluster sequences are placed in blue (these cluster with similar species), others PacBio cluster sequences placed in red (variable PacBio cluster sequences, clustering with other species/strains). All Sanger sequences of studied strains are placed in green to highlight their phylogenetic position in the tree.

As phylogenetic analyses showed that Dream Taq has a better performance in term of molecular identification that SuperFi DNA polymerase (Appendix A: Figure A1), analyses of sequence divergence between the PacBio consensus sequences and the Sanger sequences were conducted only using the PacBio sequences resulting from this polymerase. In order to characterize the intragenomic variation, average p-distance of whole nrITS (ITS1-5.8s-ITS2) sequences were calculated using MEGA6 [41] between PacBio consensus sequences of different clusters within strains. To characterize the discrepancy between the PacBio and Sanger sequences, we also calculated average p-distance between all PacBio consensus sequences and their corresponding Sanger sequences of the respective strains. Finally, to have overall insights into intraspecific variation, average p-distance was calculated between strains using either Sanger sequences or PacBio sequences of respective strains within species.

### 2.6. Haplotype Network Analysis of nrITS 

Haplotype networks for eight *Cordyceps* species (except *C. cateniannulata* which only had one PacBio consensus sequence) were constructed using PopArt v. 1.7 [42]. A Minimum Spanning Network (MSN) method was used from the aligned nrITS data set of individual species using default settings and *Ɛ* value set at 0. Augmenting the *Ɛ* value from 0 to 10 increased the pattern complexity for the interconnected node of the different grouping, which led to difficulty in interpreting the haplotype network [43,44]. Combined data (PacBio consensus sequences and Sanger sequences) were used to infer haplotype networks map presented in this study. 

## 3. Results

### 3.1. PacBio Sequencing

Following a clustering at 97% similarity, based on the data from the Dream Taq DNA polymerase, each strain contained one to seven clusters for the ITS or D1–D2 LSU regions. One dominant cluster of each strain could enclose from 99 to 427 reads, while remaining minor clusters held mostly one to 14 reads/strain. Detailed information for the inferred clusters is provided in Supplementary Information (Tables S2 and S3). The ITS region resulted generally in more clusters than the D1–D2 LSU region, supporting the idea that ITS has higher intragenomic variation than LSU. Using a high fidelity DNA polymerase (Platinum SuperFi DNA polymerase (Invitrogen)), similar numbers of cluster were obtained (Table S4). Success rate of PCR with the high fidelity DNA polymerase was around 81%, whereas the Dream Taq amplified all 22 strains.

PacBio sequencing revealed many clusters for most of the strains due to the intragenomic variation. However, some variations might be due to sequencing errors. We filtered out all reads with less than five subreads which guaranteed at least 99% accuracy. Furthermore, the obtained PacBio sequences have average read depth (number of subreads) of 32.7, corresponding to more than 99.999% accuracy. With 97% similarity of clustering and this level of sequencing accuracy, potential erroneous reads would be masked within consensus sequences and would not impact on the species identification. However, species identification based on PacBio consensus sequences can be subject to confusion with blast search in a public database, because most of the deposited sequences were obtained from the Sanger platform and might be different in length due to different bioinformatics protocol, which would impact on the accuracy of blast hits with PacBio sequences. Furthermore, as high-throughput sequencing can reveal most forms of intragenomic variants, not only the dominant type, but also minor variants with substantial divergence might match with sequences from other species. Our Blast results for all PacBio consensus sequences are summarized in the supplementary information (Tables S5 and S6) and showed that, while most of the PacBio sequences did not match with the corresponding Sanger sequences of respective strains and species in the NCBI nucleotide database, those PacBio consensus sequences with the most CCS reads did mostly match with the corresponding Sanger sequences deposited in NCBI database. 

### 3.2. Molecular Phylogeny

All sequences (Sanger and PacBio) of ITS, D1–D2 LSU and combined regions were aligned separately and datasets of 623, 877 and 1345 nucleotides were obtained respectively. The phylogenetic relationships were inferred using Bayesian analyses and maximum likelihood (ML).

**Figure 2:**
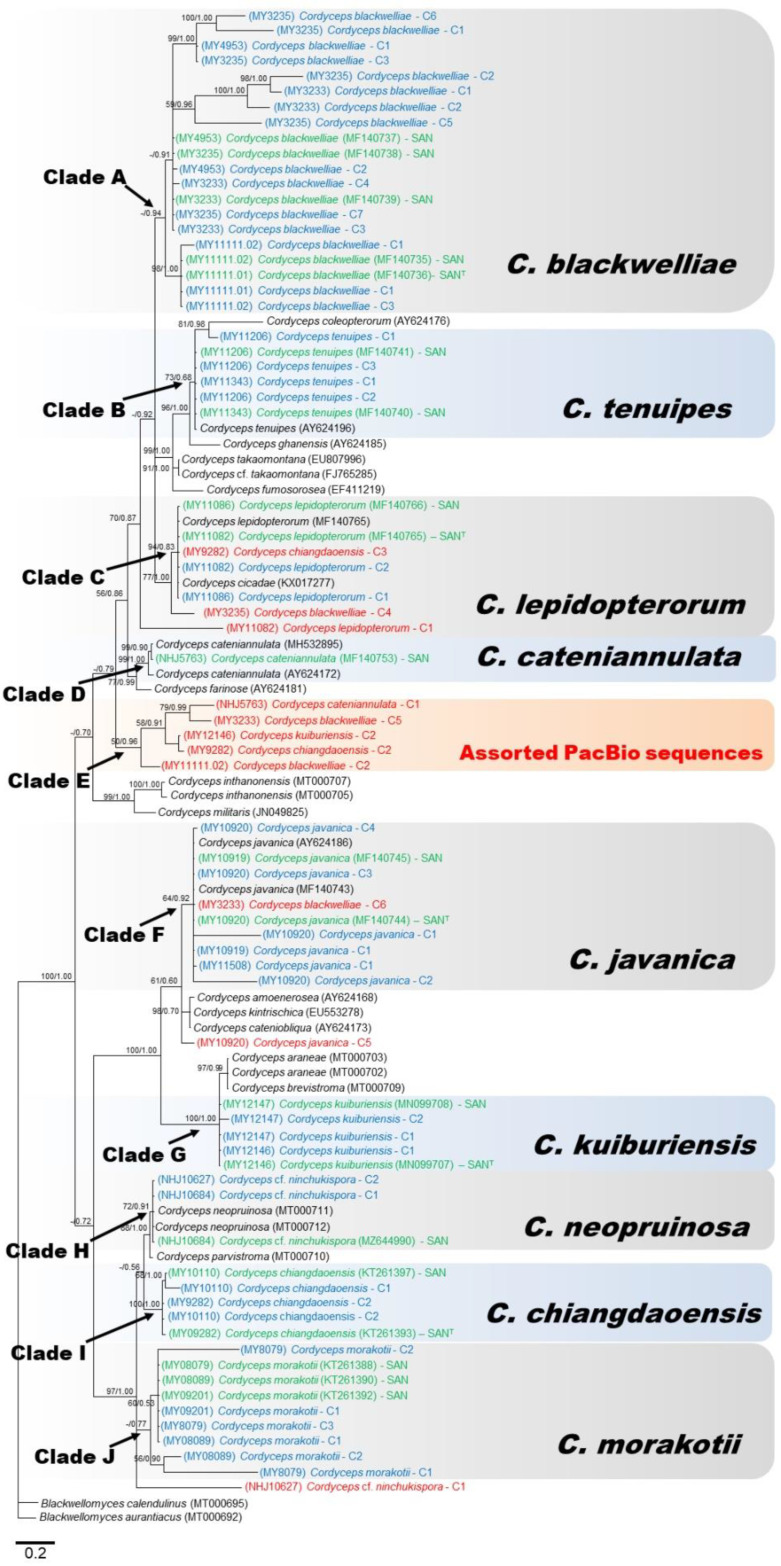
Consensus phylogram (50% majority rule) from a Bayesian analysis of the nrITS sequences, obtained from 10^6^ MCMC generations. Maximum likelihood bootstrap values >50% (left of/) and Bayesian posterior probabilities >0.50 (to the right). The scale bar represents substitution rate per site. The PacBio cluster sequences highlighted in blue are those clustered within corresponding true species clades; other PacBio cluster sequences highlighted in red are those branched outside the species of origin. All Sanger sequences of the studied strains are highlighted in green. Sanger sequences of type species demarked as ‘T’.

**Figure 3:**
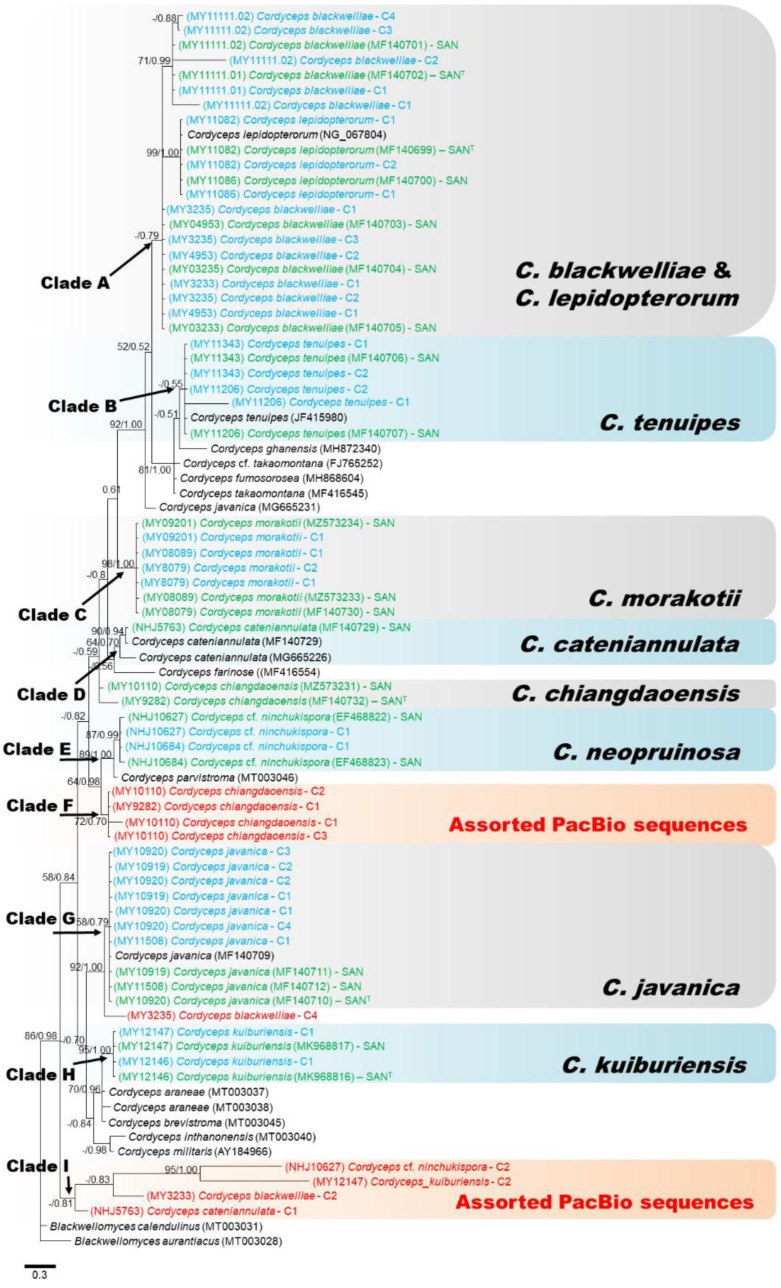
Consensus phylogram (50% majority rule) from a Bayesian analysis of the nrLSU sequences, obtained from 10^6^ MCMC generations. Maximum likelihood bootstrap values >50% (left of/) and Bayesian posterior probabilities >0.50 (to the right). The scale bar represents substitution rate per site. The PacBio cluster sequences highlighted in blue are those clustered within corresponding true species clades; other PacBio cluster sequences highlighted in red are those branched outside the species of origin. All Sanger sequences of the studied strains are highlighted in green. Sanger sequences of type species demarked as ‘T’.

**Figure 4:**
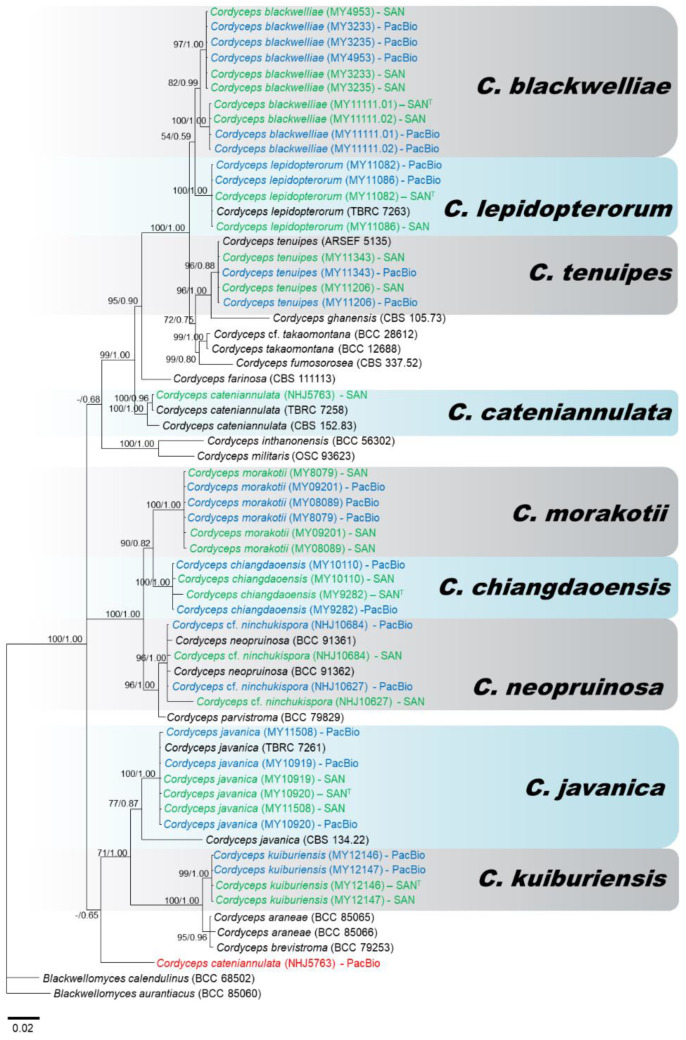
Consensus phylogram (50% majority rule) from a Bayesian analysis of the nrITS-nrLSU combined sequences, obtained from 10^6^ MCMC generations. Maximum likelihood bootstrap values >50% (left of/) and Bayesian posterior probabilities >0.50 (to the right). The scale bar represents substitution rate per site. The PacBio cluster sequences highlighted in blue are those clustered within corresponding true species clades; other PacBio cluster sequences highlighted in red are those branched outside the species of origin. All Sanger sequences of the studied strains are highlighted in green. Sanger sequences of type species demarked as ‘T’.

nrITS (Figure 2) and nrLSU (Figure 3) phylogenies could be divided into ten (A-J) and nine (A-I) clades, respectively. Combined ITS and LSU phylogeny is represented in Figure 4. ITS and LSU phylogenetic trees differed from each other but allowed overall classification into different *Cordyceps* species. However, some PacBio sequences were clearly mis-classified. The nrITS tree (Figure 2) showed Clade A representing the five strains of *C. blackwelliae* with all Sanger sequences and most of the PacBio sequences together while, in the LSU phylogeny, *C. blackwelliae* nested with *C. lepidopterorum* (Figure 3: clade A). Both ITS and LSU tree gave a glimpse of intraspecific variations in *C. blackwelliae* where strains tended to be separated into two groups (MY11111.01 and MY11111.02 vs. the others). The previous study of Mongkolsamrit et al. [27] recognized two different subclades within the *C. blackwelliae,* based on multigene phylogeny. In the ITS phylogeny, *C. lepidopterorum* was placed in clade C with *C. cicadae* (KX017277) and one PacBio sequence clustered of *C. chiangdaoensis* (MY9282-C3). An earlier study of Mongkolsamrit et al. [25] actually showed that *C. cicadae* nested together with *C. lepidopterorum*. 

All PacBio and Sanger sequences of *C. tenuipes* grouped together in both phylogenies (Clade B: Figure 2 and Figure 3), and were placed as closely related to *C. ghanensis,* as had been found in Mongkolsamrit et al. [27]. However, we can notice that an ITS PacBio sequence of MY11206 clustered with a Sanger sequence picked from NCBI of *C. coleopterorum* and revealed a certain level of intragenomic variation. Between the two strains of *C. tenuipes*, only four copies of ITS were found (one (MY11343) and three (MY11206)). Clade D of both phylogenies corresponded to *C. cateniannulata*, represented by Sanger sequences of ITS and LSU while the only ITS and LSU copy as covered by PacBio sequencing clustered with PacBio sequences from others *Cordyceps* species in a distinct clade. Clade F (Figure 2) and Clade G (Figure 3) represented *C. javanica* with the clustering of Sanger and PacBio sequences from various strains together for ITS and LSU, respectively. All LSU sequences of *C. javanica* clustered together with similar branch lengths and revealed less intragenomic variation than ITS for which most PacBio sequences clustered together, except one (MY10920-C5), and two sequences (MY10920-C1 and C2) which gave longer branches than the other sequences, due to sequence variation within the genome. Clade G of ITS phylogeny corresponded to *C. kuiburiensis*; Sanger sequences and most of the PacBio consensus sequences clustered together but were placed at the very proximity with *C. araneae* and *C. brevistroma*, while Clade H of the LSU phylogeny consisted only of sequences from this species, revealing that intra-genomic variation is higher in the ITS region. In the ITS phylogeny (Figure 2), all Sanger and PacBio sequences of *C.* cf. *ninchukispora* (except NHJ10627-C1) grouped together in Clade H with known sequences of *C*. *neopruinosa* from NCBI. The same pattern was observed in the LSU phylogeny in Clade E (Figure 3), suggesting that our *C.* cf. *ninchukispora* sequences should be re-classified as *C. neopruinosa*. Clade I of the ITS phylogeny represented two Sanger and three PacBio sequences of *C. chiangdaoensis* except MY9282-C3 which clumped with *C. lepidopterorum* (Clade C), but in the case of LSU phylogeny the Sanger and PacBio sequences clustered in different clades (Figure 3: clade F). Clade J of ITS phylogeny represents *C. morakotii*, (Figure 2) and is further divided into two subclades, one of which consisted only of PacBio sequences, while all Sanger and other PacBio sequences clustered together in Clade C of the LSU phylogeny. The combined phylogeny (using sequences with most PacBio reads) of ITS and LSU (Figure 4), showed a better resolution for all *Cordyceps* species identification except *C. cateniannulata*. 

### 3.3. Sequence Divergence Analysis

The intragenomic variation as characterized by p-distance between PacBio sequences within strains varied from 0.002 (MY4953: *C. blackwelliae*) to 0.088 (MY08079: *C. morakotii*), and was for many strains substantially higher than intraspecific variation which was characterized by p-distance between Sanger sequences of respective strains within species (Table 2). This means that PacBio sequencing revealed some divergent sequences within individual strains, outside the range of the species. This observation was also confirmed by phylogenetic analyses where some PacBio sequences were placed outside their species of origin (red highlight in Figure 2); these sequences came from strains for which the intragenomic variation exceeded intraspecific variation (Table 2). For *C. morakotii* (MY08079 and MY08089), the PacBio sequences were grouped within the species clade but long branches and distinct position revealed a notable level of intragenomic variation while all the Sanger sequences had very short branches, corresponding to the observation based on p-distance that the intragenomic variation was still higher than the intraspecific variation. 

Intragenomic variation was thus the cause of discrepancy between Sanger and PacBio sequences, as average p-distance between the two types of sequences was also higher than p-distance between strains within species based on Sanger sequences (Table 2). Some strains had no intragenomic variation with only one type of PacBio sequence (Table S1); these strains had actually zero divergence to their respective Sanger sequences (Table 2) except NHJ5736 (*C. cateniannulata*), which had one PacBio ITS type totally different from its Sanger counterpart, but this was probably due to a contamination.

### 3.4. Haplotype Network Analysis of nrITS

**Figure 5:**
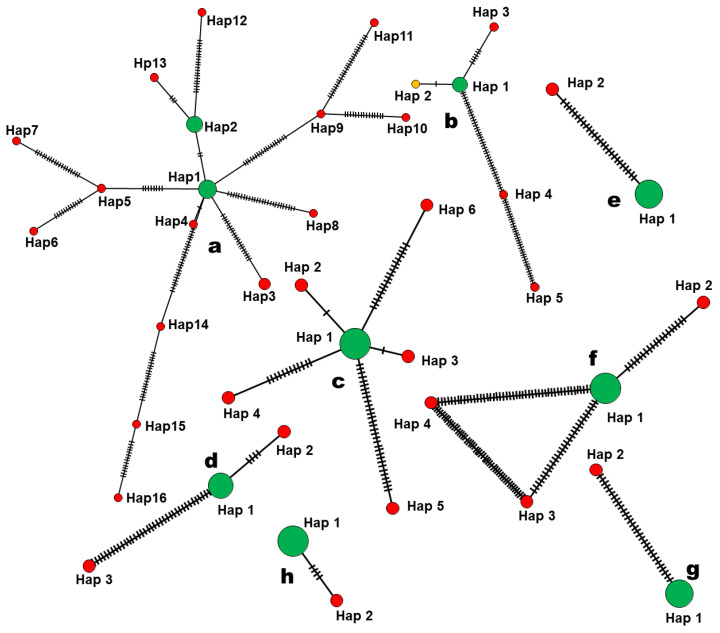
Haplotype network of *Cordyceps* species: (**a**) *C. blackwelliae*; (**b**) *C. chiangdaoensis*; (**c**) *C. javanica*; (**d**) *C. kiburiensis*; (**e**) *C. lepidopterorum*; (**f**) *C. morakotii*; (**g**) *C.* cf. *ninchukispora*; (**h**) *C. tenuipes*. Green colour represents haplotypes inferred from both Sanger and PacBio sequences; Yellow colour represents haplotypes with only Sanger sequences and red colour represents haplotypes with only PacBio sequences.

Two (*C. lepidopterorum; C.* cf. *ninchukispora;* and *C. tenuipes*) to 16 (*C. blackwelliae*) haplotypes were identified within species. In every species, we found one to two main haplotypes representing dominant variants within the genome, consisting of PacBio sequences with most sub-reads and their corresponding Sanger sequences. The main haplotypes are closely related while the minor haplotypes are always separated by several mutational steps from the main haplotypes (Figure 5). For example, in the case of *C. blackwelliae*, 16 different haplotypes with a relatively expanded network (Figure 5a) indicated high intragenomic variation with two main haplotypes (Hap 1, Hap 2) which included Sanger sequences and dominant PacBio sequences with most reads (Table 3), whereas other haplotypes were separated by many mutational steps and included PacBio sequences with very few reads (minor variants). *Cordyceps chiangdaoensis* had five different haplotypes (Figure 5b); Hap 1 and Hap 2 were closely related and separated by only one mutational step whereas Hap 5 had many mutational steps. Figure 5c represents *C. javanica* which had six different haplotypes; Hap 1, Hap 2 and Hap 3 were closely related with one mutational step while Hap 5 had many mutational steps. *C. kuiburiensis* (Figure 5d) had three different haplotypes with Hap 3 showing much distance from Hap 1. *Cordyceps lepidopterorum* and *C.* cf. *ninchukispora* (Figure 5e,g) each had only two haplotypes, one dominant and one minor, with several separating mutational steps. *Cordyceps morakotii* (Figure 5f) comprised one main haplotype and three minor haplotypes separated by several mutational steps. *Cordyceps tenuipes* (Figure 5h) had only two different relatively closely related with only four mutational steps.

## 4. Discussion

Long-read sequencing technology such as PacBio has received increasing attention for metabarcoding (identification from environmental or medical examples) [45,46]. This technology has also been used for generating molecular barcodes to well-identified herbarium specimens [47]. The major interest in the latter case is to evaluate whether long-read HT-amplicon sequencing could generate barcodes allowing accurate species identification, which is similar to the objective of our study, where we evaluated the performance in identifying *Cordyceps* species from our culture collections. Concerns were raised regarding the use of such technology in species identification. At first instance, the raw error rate of PacBio sequencing is as high as 13% to 15% [48], but rigorous bioinformatics protocol and recent methods such as circular consensus sequence (CCS), as used in PacBio SEQUEL I in our study, have contributed to a great improvement in accuracy [49]. Errors could arise also before the sequencing process through tag switching during the library preparation [46], but our approach based on dual indexing allowed detection and removal of tag-switching artefacts [50]. Intragenomic variation is a potential source of identification errors via HT-amplicon sequencing [51]. Fungal strains can be heterozygous, multi-nucleated or originate from multiple haploid spores; in these cases, different genomes found within individual strains could have divergent molecular types. This problem would be exacerbated for identification of closely related species where divergent molecular types could circulate through permeable reproductive barriers. 

The ITS and LSU phylogenies in our study showed that some PacBio sequences clustered outside their putative species. On the one hand, these sequences may represent minor variants within the genome that escaped concerted evolution, as they were represented by only one or few CCS reads within each cluster (Tables S2 and S3). On the other hand, they may be attributed to sequencing errors. In the latter case, sequencing errors are supposed to be random, generating sequences that should be randomly placed in the phylogenies, while our results clearly showed a tendency of some PacBio sequences from distinct species to clump together in a few clades (assorted PacBio sequences in Figure 2 and Figure 3). These results supported the view that genuine intragenomic variation exists in our data. We made a combined phylogeny using both regions (ITS and D1–D2 LSU), using only PacBio consensus sequences with the highest numbers of reads (Figure 4), which showed that the majority of PacBio sequences clustered with their respective Sanger sequences, forming clades including type strains (Figure 4). An exception was found for *C. cateniannulata* (NHJ5763) which only had one PacBio consensus sequence for each region; the phylogenies of these regions, either separately or combined, placed the PacBio sequences of this strain outside the true species clade. As these sequences had high coverage (192), PCR and sequencing errors seemed improbable. A contamination or an error during the processing of the strain might be the cause as the blast results for NHJ5763 (Table S5 and S6) showed that it matched with sequences from *Akanthomyces* sp., *Gibellula* and *Isaria* which are all closely related genera. More strains of *C. cateniannulata* and additional sequencing are needed to clarify this problem. Overall, using both barcodes (ITS and LSU) generated from PacBio sequencing allowed corrected identification of most of the studied species.

High-fidelity DNA polymerase has been suggested for use in high-throughput amplicon sequencing to minimize errors related to PCR amplification and standard DNA library preparation [52]. The ITS phylogeny with sequences derived from SuperFi DNA polymerase revealed surprisingly more clades consisted of sequences clustering outside their true species clades (Figure A1) than the Dream Taq DNA polymerase. These clades are likely to be due to genuine intragenomic variation, not to sequencing errors, as high-fidelity polymerase is more sensitive than standard Taq and is able to better detect true allelic variants [53,54]. The consensus sequences with maximum number of reads were nevertheless grouped with Sanger sequences of the putative species. Therefore, although high-fidelity polymerase may be better in detecting intragenomic variation, a standard Taq DNA polymerase offers a less expensive option for identification based on consensus sequences with the maximum reads. 

In the last two decades, several studies reported intragenomic variation in fungi, especially in Basidiomycota, some of them dealt with taxonomic species identifications [55,56,57,58,59]. Most of the studies were conducted through cloning combined with Sanger sequencing or Restriction Fragment Length Polymorphisms (RFLP) profiling [60]. Some Basidiomycetes fungi including *Rhizoctonia solani*, *Laetiporus* sp., and *Ogataea ovarum* were shown to extensively possess intragenomic variation that confused species identification [57,61,62]. On the other hand, several species of the genera *Amanita*, *Ceraceosorus*, *Russula*, *Boletus*, *Cortinarius*, *Cantharellus*, *Lactarius* showed very little intragenomic variation, without particular problems for species identification [55,56,58,59,63,64]. Some Ascomycetes, including *Phoma exigua*, *Magnaporthe grisea*, *Davidiella tassiana*, *Mycosphaerella punctiformis*, *Saccharomyces cerevisiae*, *Teratosphaeria microspora* showed a greater amount of intragenomic variation which affected proper species identification [65,66,67]. A recent study by Stadler et al. [68] in the family Hypoxylaceae via genome mining reported that *Hypoxylon fragiforme* and *Xylaria hypoxylon* contained 19 and 13 copies of ITS, but most of the copies were homogeneous. In contrast, our study showed that some *Cordyceps* species contained divergent rDNA copies within the genome, potentially confusing proper identification (see Figure 2). It is necessary to apply a similar approach to other groups of fungi in order to test the validity of the use of PacBio technologies to identify species based on phylogenetic classification. 

The haplotype network among the DNA sequences is useful for gaining insights into micro-evolutionary process within species and genomes. The network approach is not dependent on a specific evolutionary model [42]. The haplotype network from our data showed that some minor variants must have escaped concerted evolution and persist within genome. This analysis re-enforced what we had found with phylogenetic analyses. 

Amplicon sequencing can extract allelic variants within the genome [69]. In metabarcoding, high intraspecific (and intragenomic) variation within nrITS is highly problematic as the diversity will be overestimated by treating every haplotype as a biological entity in downstream statistical analysis [8,44]. The problematic in our study is slightly different. We are interested in knowing whether intragenomic variation could bias species identification of curated culture collections and specimens. Despite a notable level of intragenomic variation in some species studied here, the main haplotypes which generally represent the dominant variants within genome could be used for species identification under molecular phylogenetic framework.

## 5. Conclusions

Nuclear ribosomal DNA have sufficient variability that can discriminate between species. Minor variants within the genome which escaped concerted evolution can misidentify specimens into wrong species as they have accumulated too many mutations from the dominant type in genome. HT amplicon-sequence can be used, on the one hand, to study intragenomic variation by revealing the various molecular types within genome, but, on the other hand, is a source of confusion for species identifications, as shown by our study. 

We demonstrated that the intragenomic variation among *Cordyceps* species was common. The reason why some species had substantially higher intragenomic variation than others is unclear and merits further investigation. The principal challenge in using HT sequencing data for species identification is to select the right variant corresponding to true species. Cluster consensus sequences containing the most reads correspond to the major variants and can be used for accurate identification. High-fidelity DNA polymerase with its lower misincorporation rate can give a more accurate account of intragenomic variation but, by doing so, results in the uncovering of more mal-identified sequences of minor variants shared between species and gives confusing signals. PacBio consensus sequences with maximal reads represents a powerful framework for species identification. 

## Figures and Tables

**Table 1:** Strains of nine *Cordyceps* species included in this study (* = Obtained in this study; ^T^ = Type strain).

Species	Original Code	BCC Code	TBRC Code	BBH Code	ITS Accession	LSU Accession
*C. blackwelliae*	MY3233	BCC 30924	TBRC 7253	BBH 23883	MF140739	MF140705
	MY3235	BCC 30926	TBRC 7254	BBH 23885	MF140738	MF140704
	MY4953	BCC 37652	TBRC 7255	BBH 26339	MF140737	MF140703
	MY11111.01 ^T^	BCC 79714	TBRC 7256	BBH 40750	MF140736	MF140702
	MY11111.02	BCC 79855	TBRC 7257	BBH 40750	MF140735	MF140701
*C. cateniannulata*	NHJ5763	BCC 1856	TBRC 7258	-	MF140753	MF140729
*C. chiangdaoensis*	MY9282 ^T^	BCC 68469	TBRC 7274	-	KT261393	MF140732
	MY10110	BCC 75733	-	-	KT261397	MZ573231 *
*C. javanica*	MY10919	BCC 79245	TBRC 7259	BBH 40411	MF140745	MF140711
	MY10920 ^T^	BCC 79246	TBRC 7260	BBH 40412	MF140744	MF140710
	MY11508	BCC 82944	TBRC 7262	BBH 41986	MF140746	MF140712
*C. kuiburiensis*	MY12146 ^T^	BCC 90322	-	BBH 45453	MN099707	MK968816
	MY12147	BCC 90323	-	BBH 45454	MN099708	MK968817
*C. lepidopterorum*	MY11082 ^T^	BCC 79840	TBRC 7263	BBH 40735	MF140765	MF140699
	MY11086	BCC 79842	TBRC 7264	BBH 40737	MF140766	MF140700
*C. morakotii*	MY8079	BCC 55820	TBRC 7275	-	KT261388	MF140730
	MY08089	BCC 55830	-	-	KT261390	MZ573233 *
	MY09201	BCC 68403	-	-	KT261392	MZ573234 *
*Cordyceps* cf. *ninchukispora*	NHJ10627	BCC 02744	-	-	-	EF468822
	NHJ10684	BCC 02725	-	-	MZ644990 *	EF468823
*C. tenuipes*	MY11343	BCC 82079	TBRC 7267	BBH 42147	MF140740	MF140706
	MY11206	BCC 81469	TBRC 7265	BBH 41247	MF140741	MF140707

**Table 2:** Average p-distance within strain (intragenomic variation) and within the species (intraspecific variation).

Species	Strains	Intragenomic Variation (PacBio)	Sanger-PacBio Discrepancy	Intraspecific Variation (Sanger)	Intraspecific Variation (PacBio)
*C. blackwelliae*	MY3233	0.077	0.05	0.003	0.054
	MY3235	0.083	0.056		
	MY4953	0.002	0.001		
	MY11111.01	-	0		
	MY11111.02	0.053	0.025		
*C. cateniannulata*	NHJ5763	-	0.078	-	-
*C. chiangdaoensis*	MY9282	0.075	0.063	0.004	0.061
	MY10110	0.012	0.006		
*C. javanica*	MY10919	-	0	0	0.034
	MY10920	0.056	0.028		
	MY11508	-	0.002		
*C. kuiburiensis*	MY12146	0.065	0.033	0.002	0.038
	MY12147	0.007	0.003		
*C. lepidopterorum*	MY11082	0.045	0.24	0	0.03
	MY11086	-	0		
*C. morakotii*	MY8079	0.088	0.038	0	0.054
	MY08089	0.019	0.01		
	MY09201	-	0		
*C.* cf. *ninchukispora*	NHJ10627	0	-	NA	0.037
	NHJ10684	-	0		
*C. tenuipes*	MY11343	-	0	0	0.004
	MY11206	0.009	0.004		

**Table 3:** Haplotype network information of eight *Cordyceps* species. SAN = Sanger sequence; Number of reads for PacBio clusters indicated in brackets.

Species	Haplotype	Frequency	Sequences
*C. blackwelliae*	Hap 1	5	MY3233 (SAN); MY3235 (SAN); MY4953 (SAN); MY3235-C7 (341); MY4953-C1 (341)
	Hap 2	4	MY11111.01 (SAN); MY11111.02 (SAN); MY11111.01-C1 (99); MY11111.02-C3 (251)
	Hap 3	2	MY4953-C2 (2); MY3233-C3 (328)
	Hap 4	1	MY3233-C4 (1)
	Hap 5	1	MY3235-C3 (14)
	Hap 6	1	MY3235-C6 (1)
	Hap 7	1	MY3235-C1 (1)
	Hap 8	1	MY3235-C5 (1)
	Hap 9	1	MY3235-C4 (1)
	Hap 10	1	MY3233-C6 (1)
	Hap 11	1	MY3233-C5 (1)
	Hap 12	1	MY1111102-C2 (1)
	Hap 13	1	MY11111.02-C1 (1)
	Hap 14	1	MY3233-C2 (1)
	Hap 15	1	MY3233-C1 (1)
	Hap 16	1	MY3235-C2 (1)
*C. chiangdaoensis*	Hap 1	3	MY10110 (SAN); MY9282-C2 (246); MY10110-C2 (207)
	Hap 2	1	MY09282 (SAN)
	Hap 3	1	MY10110-C1 (1)
	Hap 4	1	MY9282-C1 (2)
	Hap 5	1	MY9282-C3 (1)
*C. javanica*	Hap 1	6	AY624186 (SAN); MY10919 (SAN); MY10920 (SAN); MF140743 (SAN); MY10919-C1 (241); MY10920-C3 (259)
	Hap 2	1	MY10920-C4 (1)
	Hap 3	1	MY11508-C1 (129)
	Hap 4	1	MY10920-C5 (1)
	Hap 5	1	MY10920-C2 (1)
	Hap 6	1	MY10920-C1 (1)
*C. kuiburiensis*	Hap 1	4	MY12146 (SAN); MY12147 (SAN); MY12146-C1 (210); MY12147-C1 (215)
	Hap 2	1	MY12147-C2 (2)
	Hap 3	1	MY12146-C2 (1)
*C. lepidopterorum*	Hap 1	5	MY11082 (SAN); MY11086 (SAN); MF140765 (SAN); MY11082-C2 (295); MY11086-C1 (225)
	Hap 2	1	MY11082-C1 (2)
*C. morakotii*	Hap 1	6	MY08089 (SAN); MY08079 (SAN); MY09201 (SAN); MY8079-C3 (265); MY08089-C1 (350); MY09201-C1 (211)
	Hap 2	1	MY08089-C2 (1)
	Hap 3	1	MY8079-C2 (1)
	Hap 4	1	MY8079-C1 (1)
*C.* cf. *ninchukispora*	Hap 1	5	MT000711 (SAN); MT000712 (SAN); NHJ10684 (SAN); NHJ10627-C2 (95); NHJ10684-C1(186)
	Hap 2	1	NHJ10627-C1 (1)
*C. tenuipes*	Hap 1	6	AY624196 (SAN); MY11343 (SAN); MY11206 (SAN); MY11343-C1 (115); MY11206-C2 (124); MY11206-C3 (303)
	Hap 2	1	MY11206-C1 (1)

## Data Availability

The raw PacBio reads were deposited at Mendeley Data (doi:10.17632/t8sxbj4gpw.2) [35]. All newly obtained Sanger sequences were deposited at the NCBI Nucleotide database with the accession numbers shown in the Table 1 in the main text.

## Supplementary Materials

The following are available online at https://www.mdpi.com/article/10.3390/jof7090767/s1, Table S1: ITS and LSU combined phylogeny data sheet represent accession number, PacBio consensus cluster number and NCBI accession number for ITS and LSU regions, Table S2: The characteristics of PacBio clusters inferred based on 97% similarity among reads for the ITS region for each strain of *Cordyceps* species included in this study, Table S3: The characteristics of PacBio clusters inferred based on 97% similarity among reads for the D1–D2 LSU region for each strain of *Cordyceps* species included in this study, Table S4: Number of PacBio clusters inferred based on 97% similarity of ITS and D1–D2 LSU PacBio reads using Dream Taq polymerase (standard) or Platinum SuperFi DNA Polymerase (high fidelity), Table S5: NCBI blast results against ITS gene, Table S6: NCBI blast results against LSU gene.
